# Emotional responses to Hindustani *raga* music: the role of musical structure

**DOI:** 10.3389/fpsyg.2015.00513

**Published:** 2015-04-30

**Authors:** Avantika Mathur, Suhas H. Vijayakumar, Bhismadev Chakrabarti, Nandini C. Singh

**Affiliations:** ^1^Speech and Language Laboratory, Cognitive Neuroscience, National Brain Research CentreManesar, India; ^2^Centre for Integrative Neuroscience and Neurodynamics, School of Psychology and Clinical Language Sciences, University of ReadingReading, UK

**Keywords:** music, emotion, *ragas*, rhythmic regularity, tempo, tonality

## Abstract

In Indian classical music, *ragas* constitute specific combinations of tonic intervals potentially capable of evoking distinct emotions. A *raga* composition is typically presented in two modes, namely, *alaap* and *gat*. *Alaap* is the note by note delineation of a *raga* bound by a slow tempo, but not bound by a rhythmic cycle. *Gat* on the other hand is rendered at a faster tempo and follows a rhythmic cycle. Our primary objective was to (1) discriminate the emotions experienced across *alaap* and *gat* of *ragas*, (2) investigate the association of tonic intervals, tempo and rhythmic regularity with emotional response. 122 participants rated their experienced emotion across *alaap* and *gat* of 12 *ragas*. Analysis of the emotional responses revealed that (1) *ragas* elicit distinct emotions across the two presentation modes, and (2) specific tonic intervals are robust predictors of emotional response. Specifically, our results showed that the ‘minor second’ is a direct predictor of negative valence. (3) Tonality determines the emotion experienced for a *raga* where as rhythmic regularity and tempo modulate levels of arousal. Our findings provide new insights into the emotional response to Indian* ragas* and the impact of tempo, rhythmic regularity and tonality on it.

## Introduction

While music has long been associated with emotions (Miller and Williams, 2008; Patel, 2010), it has also been a subject of interesting debate among philosophers. Consequently, the existence of emotions induced by music has been debated by believers and non-believers referred to as emotivists and cognitivists, respectively. The cognitivists argue that music does not generally evoke emotions in listeners, it merely expresses emotions that are perceived by listeners (Kivy, 1989). In other words, listeners refer to music as happy or sad because the music expresses happiness or sadness, not because the music makes them feel happy or sad. By contrast, emotivists suggest that music actually evokes or induces feelings in listeners (Scherer and Zentner, 2001). Recent studies that have focused on measures other than self reports, namely changes in arousal levels measured by changes in autonomic nervous system activity while listening to music (Krumhansl, 1997; Nyklíček et al., 1997), indicate that music does evoke emotions (for review refer to Sloboda and Juslin, 2010). As a result, many theories have been put forward to explain the mode of induction of emotions by music. Emotional reactions to music have been explained in terms of cognitive appraisals, which claim that emotions are elicited or differentiated on the basis of an individual’s subjective evaluation or appraisal (Scherer, 1999). More recently, Juslin and Västfjäll (2008) have argued that cognitive appraisals are only one of the ways in which emotions are induced, and have proposed six other mechanisms that explain how musical pieces induce emotions: (1) brain stem reflexes (e.g., reactions to dissonance), (2) conditioning (i.e., a particular music is associated with a positive or negative emotion), (3) contagion (i.e., listener perceives the emotional expression of music, and then “mimics” this expression internally), (4) visual imagery (i.e., images evoked by music act as cues to an emotion), (5) episodic memory (i.e., a piece is associated with a particular event, which, in turn, is associated with an emotion), and (6) expectancies that are fulfilled or denied (i.e., emotion is induced in a listener because a specific feature of the music violates, delays, or confirms the listener’s expectations about the continuation of the music).

In the research reported here, we used self-reports by participants as a measure to study the subjectively experienced feeling of emotion while listening to *ragas* of North Indian Classical music (NICM). NICM born out of a cultural synthesis of the Vedic chant tradition and traditional Persian music has been known to induce emotions (Kaufmann, 1965). The central notion in this system of music is that of a *raga*. The word ‘*raga*’ originates in Sanskrit and is defined as ‘the act of coloring or dyeing’ (the mind and mood/emotions in this context) and therefore refers metaphorically to ‘any feeling or passion especially love, affection, sympathy, desire, interest, motivation, joy, or delight.’ Thus, a *raga* composition comprises of a specific combination of notes which are used by the performer to create a mood (*rasa*) or atmosphere that is unique to the *raga*.

An extensive body of ancient Indian scripts belonging to the early centuries A.D. have documented the emotions associated with *ragas* (Bhatkhande, 1934; Natyashastra by Bharata translated by Vatsyayan, 1996). These are as follows – love, laughter, anger, compassion, disgust, horror, heroic, wonder, peace, and spiritual devotion. From a research perspective, Zentner et al. (2008) have incorporated the type of emotions elicited while listening to music into a scale – the Geneva Emotional Scale (GEMS) which are labeled as wonder, transcendence, tenderness, peacefulness, nostalgia, power, joyful entertainment, tension, and sadness. We used a combination of these two sources in order to arrive at the emotion labels used for this study, namely, happy, romantic, devotional, calm/soothed, angry, longing/yearning, tensed/restless, and sad.

The basic set of tones and tone-relationships used in NICM from which *ragas* are derived are the 12-tone octave divisions (Castellano et al., 1984; Bowling et al., 2012). Each interval is a tone defined by the ratio of its fundamental frequency with the tonic, or ‘root’ note and is termed as tonic interval. The “major” intervals are the *shuddh swaras* or the natural notes namely, second, third, sixth, and seventh while the “minor” intervals are the *komal swaras* (flat) positions of the same tones. (Tonic interval names used in NICM, frequency ratios, sizes in cents in Just intonation and 12-tone equal temperament (12-TET) tunings and the corresponding interval name in the Western chromatic scale are provided in **Table 1**). Apart from the 12 tones mentioned in **Table 1**, there exist a set of 10 more intermittent tones which comprise the 22 *sruti* system in Indian classical music (Loy, 2011). *Sruti* refers to subtle intervals produced because of oscillations in pitch. This occurs when a note is subjected to a slow shake or an exaggerated vibrato, either as a decoration or as a functional feature of a *raga*. However, the prevalence of the 22 *srutis* in the modern period is a subject of much discussion and debate. A recent analysis of *srutis* by Serra et al. (2011) has shown that Hindustani classical music has equal-tempered influences as compared to Carnatic music which emphasizes on ornamentation (Koduri et al., 2012) and follows the Just-Intonation system. Consequently, we used the 12-tone classification in Equal temperament scale for evaluating the tonality of* ragas*.

**Table 1:** Music intervals in Hindustani classical music.

Interval name	Abbreviation used	Western scale (Interval name)	Frequency ratio	Just intonation (Cents)	12-TET (Cents)
*Shadja*	*Sa*	Perfect unison	1	0	0
*Komal Rhishabha*	*re*	Minor second	16/15	112	100
*Shuddha Rhishabha*	*Re*	Major second	10/9	183	200
*Komal Gandhara*	*ga*	Minor third	6/5	316	300
*Shuddha Gandhara*	*Ga*	Major third	5/4	386	400
*Madhyama*	*Ma*	Perfect fourth	4/3	498	500
*Tivra Madhyama*	*ma*	Tritone	45/32	590	600
*Panchama*	*Pa*	Perfect fifth	3/2	702	700
*Komal Dhaivata*	*dha*	Minor sixth	8/5	814	800
*Shuddha Dhaivata*	*Dha*	Major sixth	5/3	884	900
*Komal Nishada*	*ni*	Minor seventh	9/5	1018	1000
*Shuddha Nishada*	*Ni*	Major seventh	15/8	1088	1100
*Shadja*	*Sa’*	Perfect octave	2	1200	1200

*Each interval is a tone defined by the ratio of its fundamental frequency to the tonic (Sa). Interval names, abbreviations used, frequency ratios and sizes in cents in Just intonation and 12-TET tunings are given in table. The corresponding interval name in the Western chromatic scale is also given. In the notation used the seven Shuddha swaras are denoted by capital letters (Sa, Re, Ga, Ma, Pa, Dha, Ni), four komal swaras, and one tivra swara are denoted by small letters (re, ga, ma, dha, ni).*

A *raga* uses a set of five or more notes from the fixed scale of seven notes, to construct a melody. However, it is not enough to define a *raga* in terms of mode or scale alone, as a number of *ragas* have the same notes, yet each maintains its own musical identity. For instance, both *ragas* *Miyan ki Malhar* and *Bahar* contain the same notes (*Sa*,* Re*,* ga*, *Ma*,* Pa*,* Dha*,* ni*, *Ni*) and yet sound quite different because of the way the notes in the scale are approached and combined. As described by Jairazbhoy (1995) when different performances of the same *raga* are examined we find that allowing for divergence of tradition and the possibility of experimentation, not only are the same notes consistently used, but also particular figurations or patterns of notes occur frequently. The most characteristic patterns of notes in a *raga* are described as ‘*pakar,*’ a catch phrase by which the *raga* can be easily recognized. These patterns of notes for a *raga* can be described in terms of their melodic movements, ascending (*aaroh*) and descending (*avroh*) lines of a *raga*. *Ragas* can have different rules of ascent and descent (for example, in *raga Desh* the ascent is a step by step pentatonic movement (*Sa*, *Re*, *Ma Pa*, *Ni Sa’*) while the descent is hepatonic (*Sa’ ni Dha Pa*, *Dha Ma Ga*, *Re Ga Sa*). Moreover, in a *raga*, in theory, two notes are given greater importance than the others. These notes are called the *vadi* – sonant, and the *samvadi* – consonant. The *vadi* is the most important note in the characteristic phrase (*pakar*) of that *raga* and is superabundant in that *raga*. On the other hand as compared to the *vadi*, the *samvadi* is described as the note that is less frequent but more than the other notes in the *raga*. The *vadi* and *samvadi* could naturally fluctuate, depending on whether the ascending or descending disjunct segments are being emphasized. For instance, in *raga Yaman*, *Ga* and *Ni* would qualify as the two most important notes in the ascent (*ni Re Ga* and *ma Dha Ni*) where as *Pa* and *Re* would be the two most important notes in the descent (*Sa’ Ni Dha Pa* and *Pa Ma Ga Re*).

A *raga* composition is typically presented as a specific sequence of events, namely the *alaap* followed by the *gat*. *Alaap* is the note by note delineation of a *raga* bound by a slow tempo, but not bound by any rhythmic cycle. *Gat* is the composition rendered at a faster tempo with accompaniment of a percussion instrument that provides a rhythmic cycle. The rhythmic cycle is measured in terms of time units or beats. These rhythmic structures can vary in the degree of pulse clarity. Pulse clarity is the estimate, on a large time scale, of how clearly the underlying pulsation in music is perceivable and is regarded as a measure for the underlying periodicity of the music (Lartillot et al., 2008). Thus, pulse clarity provides a measure of rhythmic regularity. Besides features such as pulse clarity, tempo is an important factor contributing to the perception of rhythm, which can be estimated as the number of notes presented per second. For the purpose of this study, rhythmic regularity was determined by estimating pulse clarity while tempo was determined in terms of note density of *raga* excerpts.

A review of the literature indicates that a few studies have investigated the proposal that different *ragas* express emotions that are perceived by the listener’s (Balkwill and Thompson, 1999; Chordia and Rae, 2008; Wieczorkowska et al., 2010). The earliest of these was conducted by Balkwill and Thompson (1999) where they asked 30 Western listeners to judge the expression of 12 Hindustani *ragas* intended to express anger, joy, peace, and sadness. They found that despite being culturally unfamiliar, listeners were sensitive to the intended expression of the *ragas*. A similar study was conducted by Chordia and Rae (2008) in which they studied emotional responses to five *ragas* on a scale of six emotions – happy, peaceful, sad, longing, tense, and romantic. While their results also suggested that *ragas* do consistently elicit specific emotions that are associated with musical properties, they also indicated that the primary predictors of emotion of *ragas* are pitch-class distribution, pitch-class dyad entropy, overall sensory dissonance, and note density. The multiple regression analysis conducted to determine the most important factors and their total predictive value revealed that these features in combination explained between 11% (peaceful) and 33% (happy) of response variance. However, none of the studies have elucidated the role of any specific tonic interval. To summarize, while the studies described above have clearly confirmed that distinct *ragas* elicit distinct emotions, they have used as stimuli the introductory section of *ragas* namely, the *alaap*. None of them have investigated the emotions experienced during the *gat* of *ragas*. Consequently, there is little information about the complex interplay of rhythmic regularity and tempo in predicting the emotion experienced for *gat* of *ragas*.

The current study builds on this past research and extends it to address new questions. The specific objectives of this study were to (1) discriminate the emotion experienced by *alaap* and *gat* for various *ragas* (2) investigate the effects of (a) rhythmic regularity, (b) tempo and (c) tonality, on the emotions experienced. Listener responses were sought from a diverse population, for which a website (http://emotion-in-music.nbrc.ac.in/p1/) was developed and the study was conducted online. After analyzing the emotional responses, a label of emotion experienced was assigned to each* raga*. Three musical features, namely, pulse clarity, tempo, and tonality were estimated for each* raga* composition. Our specific hypotheses were the following (1) distinct emotional responses would be associated with *alaap* and *gat* of a *raga*; (2) rhythmic regularity and tempo would both modulate emotional response. (3) Since the emotion associated with a *raga* is believed to be an attribute of the tonic intervals from which it is derived, tonality would influence the emotional response.

## Materials and Methods

Three minute instrumental renditions of 12 *ragas* were played by a professional musician on *sarod* (a stringed instrument) and digitally recorded in both *alaap* and *gat*. Participants were permitted to provide emotion ratings only after listening to the composition for at least 1 min. This had two advantages: (a) it ruled out random responses, and (b) it gave each participant the flexibility to listen to the composition as per their choice between 1 and 3 min. The list of the *ragas* played and scale used by the artist are given in **Table 2**. All the pieces in *gat* were provided similar rhythmic accompaniment on *tabla* in *teen taal*, a rhythm symmetrical in structure having sixteen beats in four equal divisions. One-minute sample in both *alaap* and *gat* are given as supplementary files (see Supplementary audio clips S1, S2, S3, and S4). A short cartoon film was shown to the participants in an attempt to ensure that all the participants began the survey in a pleasant mood. Participants were instructed to listen to *raga* excerpts for a minimum of 1 min and rate each *raga* on all the following emotions on a 0–4 Likert scale (with 0 being *not at all felt’* to 4 being ‘*felt the most’*). The emotion labels were; happy, romantic, devotional, calm/soothed, angry, longing/yearning, tensed/restless, and sad. The emotion labels in the response form were also presented in Hindi and transliterated to Hindi (for example, Happy – -4pt, khush). The *ragas* were presented in alternating *alaap* and *gat* blocks. The experiment consisted of four such blocks with each block consisting of six *ragas*. The presentation of *alaap* or *gat* as the first block was counterbalanced across subjects. The order of presentation of *ragas* within each block was randomized across participants. The participants were given an option to opt out of the survey after rating atleast two blocks (i.e., 12 *ragas* – six *alaap* and six *gat*). The survey was presented in English.

**Table 2:** The table lists the *ragas* used in the study and the scale used by the artist to play the *raga*.

S.No.	*Raga*	Scale used by Artist						
1	*Tilak kamod*	*Sa*	*Re*	*Ga*	*Ma*	*Pa*	*Dha*	*Ni*
2	*Hansadhwani*	*Sa*	*Re*	*Ga*		*Pa*		*Ni*
3	*Desh*	*Sa*	*Re*	*Ga*	*Ma*	*Pa*	*Dha*	*ni/Ni*
4	*Yaman*	*Sa*	*Re*	*Ga*	*ma*	*Pa*	*Dha*	*Ni*
5	*Rageshree*	*Sa*	*Re*	*Ga*	*Ma*		*Dha*	*Ni*
6	*Jog*	*Sa*		*ga/Ga*	*Ma*	*Pa*		*Ni*
7	*Marwa*	*Sa*	*re*	*Ga*	*ma*		*Dha*	*Ni*
8	*Lalit*	*Sa*	*re*	*Ga*	*Ma/ma*		*dha*	*Ni*
9	*Malkauns*	*Sa*		*Ga*	*Ma*		*dha*	*Ni*
10	*Shree*	*Sa*	*re*	*Ga*	*ma*	*Pa*	*dha*	*Ni*
11	*Basant Mukhari*	*Sa*	*re*	*Ga*	*Ma*	*Pa*	*dha*	*Ni*
12	*Miyan ki todi*	*Sa*	*re*	*Ga*	*Ma/ma*	*Pa*	*dha*	*Ni*

### Participant Details

Participants were recruited through word of mouth and social media platforms. Since the study was conducted online participants from across the world participated in the study. In view of the primary objectives of the study as described earlier, the analysis presented in this study focuses only on data from Indian participants who completed atleast half the survey [i.e., rated at least six *alaap* excerpts (out of 12) and six *gat* excerpts (out of 12)]. Thus, ratings from 122 participants (*F* = 66, *M* = 56) were considered for analysis presented herewith. Their ages were distributed as follows – below 20 years (12%), 20–40 years (59%), 40–60 years (26%), and above 60 years (2%). Participants also rated their familiarity with NICM on a scale of 0–4 (0 not at all, 1 a little, 2 somewhat, 3 very, and 4 expert). Analysis of demographic details showed that 42% of the participants reported themselves as not at all or a little familiar with NICM and 56% of participants considered themselves as somewhat, very familiar or expert in NICM. Two participants (2%) did not give their familiarity details. The Institutional Human Ethics Committee of the National Brain Research Centre, India, approved the study.

### Data Analysis

Analysis was conducted at three levels (1) behavioral analysis of emotional response, (2) extraction of musical features of *ragas* and (3) correlation and regression analysis to investigate the relationship between musical features and emotional response.

The results of the survey were analyzed using SPSS v. 20 as described below.

#### Behavioral Analysis

Median ratings for each emotion were computed to assign an emotion label to a *raga*. The emotion with the highest median rating for a given *raga* was assigned as the typical emotion elicited by that *raga*.

#### Assessment of Musical Structure

##### Tempo, Rhythmic Regularity and Tonality

As per the objectives of this study, the effect of three musical structures namely tempo, rhythm and tonality on emotional response were assessed.

Tempo was estimated in terms of number of notes presented per second and was measured in terms of note density. Rhythmic regularity was measured in terms of time units or beats and was calculated in terms of pulse clarity. Matlab-based toolbox (MIR v.1.5) developed by Lartillot et al. (2008) was used to estimate both note density and pulse clarity. To estimate the note density, the mireventdensity function was used which estimates the average frequency of events (note onsets per second) for an excerpt. Similarly, pulse clarity was estimated by using mirpulseclarity function in terms of the Shannon entropy of the fluctuation spectrum of a particular musical composition (Pampalk et al., 2002). Music with easily perceived beats has a distinct and regular fluctuation spectrum and consequently has a low fluctuation entropy and high pulse clarity.

The third musical structure, namely tonality, is a central organizing principle in many different kinds of music and pitches are heard in relation to a tonic pitch (Chordia and Rae, 2008). It was calculated by estimating the mean frequency of occurrence of different tonic intervals as described by Bowling et al. (2012).

Tonic interval is the difference in cents between the fundamental frequencies of the note being compared with the tonic. The pitch was extracted using Melodia- Melody extraction toolbox for every 30 ms window for all *ragas* (Salamon and Gómez, 2012) and converted into cents using the following formula (refer to equation no. 1, f1 is the frequency of the note in Hz and f0 is the frequency of the tonic in Hz).

$$Equation\,\;1.\,\;\;\;\;\;\;\;\;\;Pitch(cents)=1200_{*}[\log_{2}(\frac{f1}{f0}\;]$$

An important point for consideration here was bin size. As pointed out in the introduction, apart from the 12 tones mentioned in **Table 1**, there exist a set of 10 intermittent tones which comprise the 22 *sruti* system in Indian classical music (Loy, 2011). Consequently, a smaller bin size would be considered more suitable to faithfully capture all the tonic intervals. However, recent work by (Koduri et al., 2012) has shown that Hindustani music uses the equi-tempered scale as compared to Carnatic music and has primarily equal-tempered influences. It is therefore sufficient to use the 12-tone classification in Equal temperament scale for evaluating the tonality of* ragas*. As seen from **Table 1**, a bin size of 100 cents would be sufficient. Accordingly, to estimate tonality, the corresponding interval size data was collated in 100 cent bins spanning three octaves (labeled from -1200 to 2400 cents). The mean frequency of occurrence of tonic intervals was calculated for each bin. Three octaves were then folded into one by adding the mean frequency of occurrence of the notes in each of the corresponding bins across the three octaves. For instance, the mean frequency of occurrence of *komal re* would be the additive mean frequency of occurrence in -1100, 100, and 1100 cent bins [refer to Supplementary Figure S1 (Image 1)].

#### Relationship Between Musical Structure and Emotional Response

To assess whether there were statistically significant differences in rhythmic regularity (pulse clarity) and tempo (note density) among the *ragas* with different experienced emotions one-way ANOVAs were conducted. The values of pulse clarity and note density for both* alaap* and *gat* of *ragas* were taken as dependent variables and the emotions experienced were taken as the independent variable.

To study the effect of tonality on emotional response, correlation analysis was conducted. Correlations were calculated between the average rating of an emotion and the mean frequency of occurrence of tonic intervals across the 12 *ragas* played in *gat*. To characterize which of these tonic intervals were the best predictors of the emotional response stepwise linear regression analysis was conducted. In the regression analysis, the vector containing average ratings for an emotion across the 12 *ragas* was taken as the dependent variable and the mean frequency of occurrence of the 12 tonic intervals was taken as the independent variable.

## Results

### Behavior

In order to assign an emotion label to a *raga* median ratings for each emotion were computed. Shapiro–Wilk normality test were conducted to assess the normality of the data. The normality tests conducted on the ratings of each emotion for all the *ragas* were significant (*p* < 0.001) indicating non-normal distributions of ratings. Consequently, non-parametric statistical tests were used to compare the median ratings of emotions for each *raga*. To evaluate differences in the medians of ratings of the eight emotions for each *raga*, Friedman one-way ANOVA by rank tests were conducted (refer to Supplementary Tables S1 and S2). The results of Friedman ANOVA were significant at *p* < 0.001. To further assess the highest experienced emotion *post hoc* Wilcoxon tests were conducted. For each *raga*, seven post-hoc Wilcoxon tests were conducted, wherein the median of the highest rated emotion was compared with other seven emotions. In the *post hoc* test, emotions whose median ratings did not differ significantly from each other [marked with an asterisk (^∗^) in Supplementary Tables S1 and S2] were considered as the highest experienced emotions by the participants for that particular *raga*. On this basis, the highest experienced emotion was determined and emotion label was assigned to each *raga*. The highest experienced emotions were, ‘calm’ and ‘sad’ for the arrhythmic phase (*alaap*) of *ragas* and ‘happy,’ ‘tensed,’ and ‘longing’ for the rhythmic phase (*gat*) of *ragas.* The *ragas* with emotion labels of calm/happy were *Hansdhwani, Tilak Kamod, Desh, Yaman, Ragesree, Jog* while *ragas* with emotion labels of sad/longing/tensed were *Malkauns, Shree, Marwa, Miyan ki Todi, Basant Mukhari, Lalit*.

**FIGURE 1:**
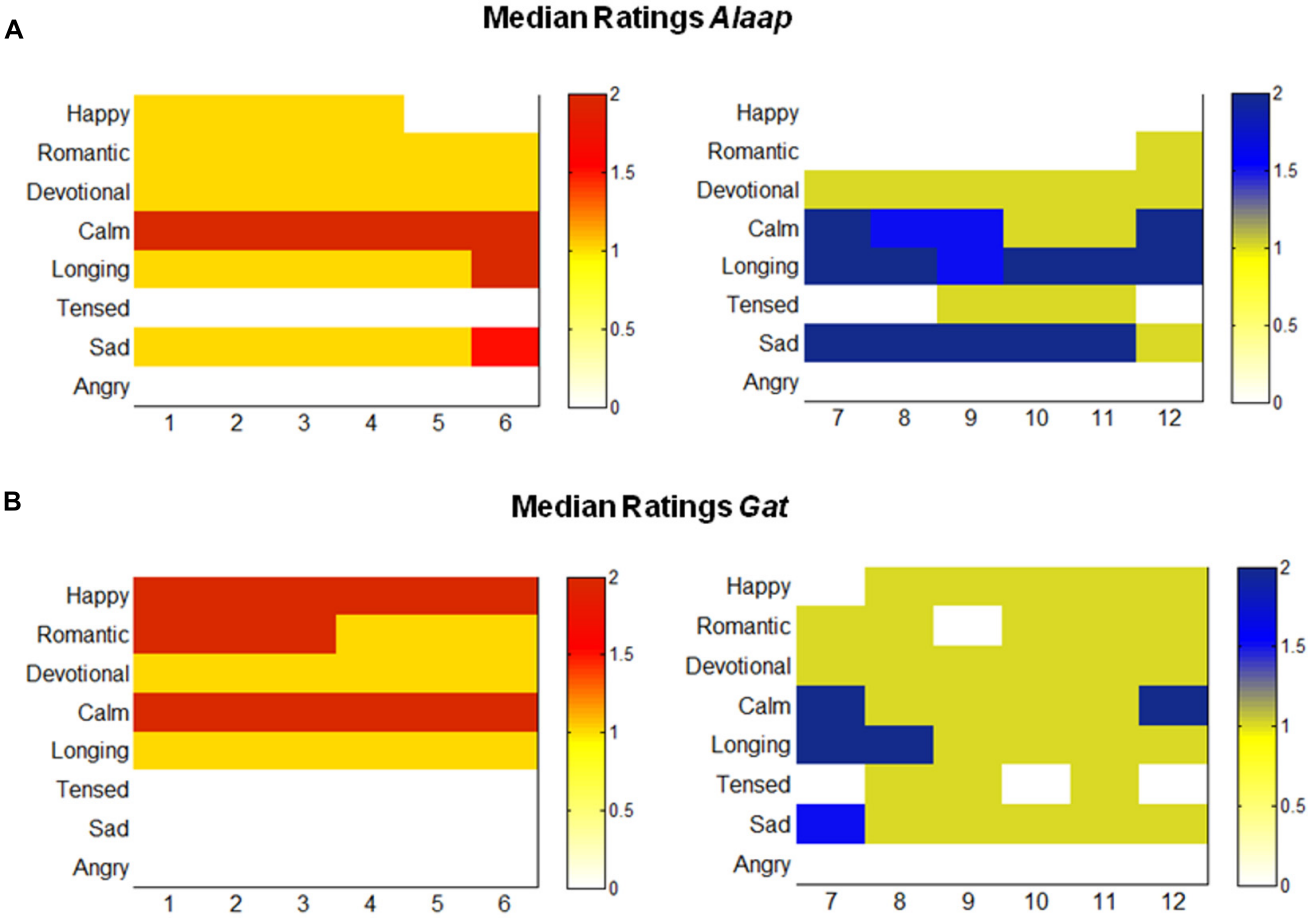
**The response matrices represent the median ratings for *ragas* across the two presentation modes (A) *alaap* and (B) *gat***. The median ratings of emotion are color coded: red represents *ragas* associated with ‘calm’ or ‘happy’ emotional response while blue represents *ragas* associated with ‘sad,’ ‘longing,’ or ‘tensed’ emotional response. The intensity of color in the color bar represents the valence of the ratings. The numbers below each matrix are representative of the following *ragas*: (1) *Hamsadhwani*, (2) *Tilak kamod*, (3) *Desh*, (4) *Rageshree*, (5) *Jog*, (6) *Yaman*, (7) *Malkauns*, (8) *Marwa*, (9) *Basant Mukhari*, (10) *Lalit*, (11) *Shree*, (12) *Miyan ki Todi*.

Response matrices representing the median ratings of experienced emotions by the participants were plotted for *alaap* and *gat* (refer to **Figure 1**). The median ratings of emotion are color coded where the intensity of color represents the strength of the emotional response. The highest median rating for *ragas* rated as ‘calm/soothing’ during ‘*alaap*’ shifted to ‘happy’ when played in *gat*. On the other hand, the highest median rating for *ragas* rated as ‘sad’ shifted to ‘longing/yearning’ or ‘tensed/restless’ during *gat*. ‘Angry’ remained the lowest rated emotion for both categories of *ragas*.

### Musical Structure

#### Rhythmic Regularity and Tempo

**FIGURE 2:**
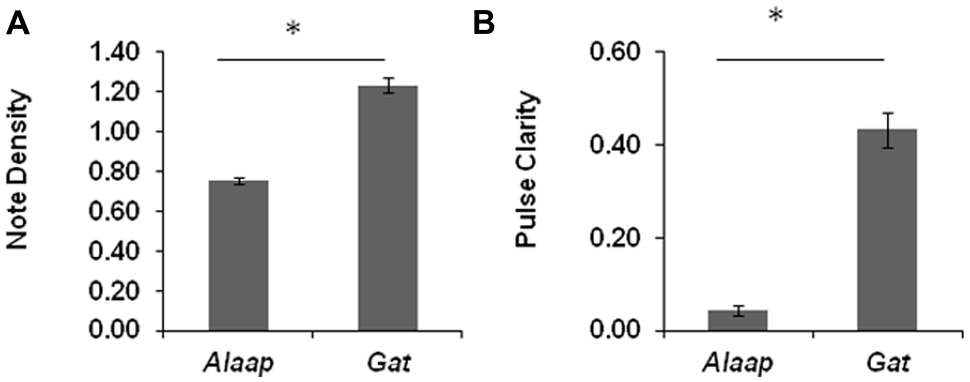
**Tempo and Rhythmic regularity**. The figure shows the average value of note density (estimate of tempo) estimated as the number of note onsets per second **(A)** and pulse clarity (estimate of rhythmic regularity) estimated as the level of rhythmic periodicities **(B)** across *alaap* and *gat* of *ragas*. Note: The paired sample *t*-tests showed that note density and pulse clarity were significantly higher for gat of ragas as compared to *alaap* at *p* < 0.001 (marked with an asterisk (^∗^)).

The average note density was higher in *gat* than in *alaap* of *ragas* (refer to **Figure 2A**). A paired sample *t*-test was conducted to compare the note density in *alaap* and *gat* of *ragas*. There was a significant difference in note density across *alaap* (*M* = 0.76, SD = 0.06) and *gat* (*M* = 1.23, SD = 0.13); *t*(11) = -13.98, *p* < 0.001. The pulse clarity was also significantly higher in *gat* as compared to *alaap* (refer to **Figure 2B**). A paired sample *t*-test conducted to compare the pulse clarity across* alaap* (*M* = 0.04, SD = 0. 01) and *gat* (*M* = 0. 43, SD = 0.04) was significant; *t*(11) = -34.76, *p* < 0.001.

Thus tempo (measured in terms of note density) and rhythmic regularity (measured in terms of pulse clarity) were both significantly higher for *gat* as compared to *alaap* of a *raga*.

#### Tonality

**FIGURE 3:**
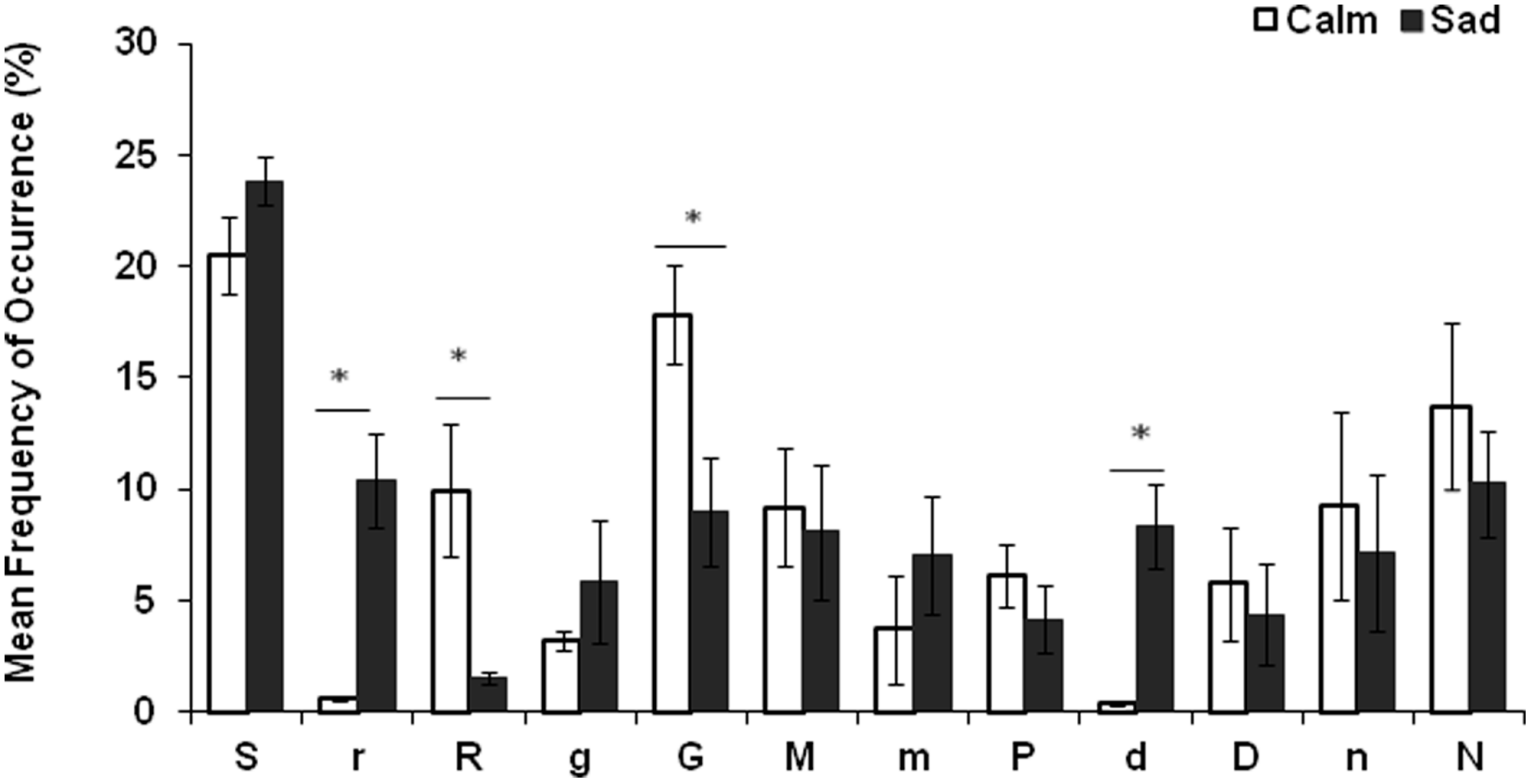
**The tonic intervals of *ragas***. The percent mean frequency of occurrence of tonic intervals averaged across *alaap* of *ragas* for which experienced emotion was ‘calm’ and ‘sad.’ Note: Two-tailed Mann Whitney *U*-test showed that percent mean frequency of occurrence of *komal re* and *komal dha* were significantly higher for *ragas* with ‘sad’ emotional response at *p* < 0.05 (marked with an asterisk (^∗^)). In addition, percent mean frequency of occurrence of *shuddh Re* and *shuddh Ga* were significantly higher for *ragas* with ‘calm’ emotional response at *p* < 0.05 (marked with an asterisk (^∗^)).

The percent mean frequency of occurrence of tonic intervals were averaged across *alaap* of *ragas* for which emotional response was ‘calm’ and ‘sad’ (**Figure 3**). The analysis of tonic intervals revealed that *ragas* that were rated for ‘calm’ were characterized primarily by major intervals (*shuddh swaras*) while those rated for ‘sad’ were characterized by minor intervals (*komal swaras*). Two-tailed Mann–Whitney *U*-test was conducted to assess the statistical significance of the differences in the mean frequency of occurrence of major and minor intervals. The results revealed that (a) the mean frequency of occurrence of the major second [*shuddh Re* (*z* = -1.92, *p* ≤ 0.05)] and major third [*shuddh Ga* (*z* = -2.24, *p* < 0.05)] was significantly higher in *ragas* with ‘calm’ emotional response. (b) The mean frequency of occurrence of minor second [*komal re* (*z* = -2.88, *p* < 0.05)] and minor sixth [*komal dha* (*z* = -2.88, *p* < 0.05)] was significantly higher for *ragas* with ‘sad’ emotional response (for complete statistics of the texts refer to Supplementary Table S3). The results remained consistent for *gat* of *ragas* [see Supplementary Figure S2 (Image 2)].

### Relationship Between Musical Structure and Emotional Response

#### Effect of Rhythmic Regularity and Tempo

The next analysis focused on investigating the relationship between emotional response and musical structure. Two separate ANOVA’s were conducted. In the first ANOVA, pulse clarity was taken as the dependent variable while the emotions experienced across *ragas* (calm, happy, sad, and tensed) were treated as the independent variable. In the second ANOVA, note density was the dependent variable, while the emotions experienced was the independent variable. The results of the two ANOVAs are summarized in **Table 3**.

**Table 3:** Results of One way Analysis of Variance (ANOVA) conducted separately to investigate whether *ragas* with different experienced emotions differ in rhythmic regularity (pulse clarity) and tempo (note density) are listed below.

Dependent variable	Independent variable: emotions experienced				
	Calm	Happy	Sad	Tensed	Significance
Note density	0.78 (0.08)	1.28 (0.13)	0.73 (0.02)	1.18 (0.11)	Welch’s *F*(3,8.78) = 59.11, *p* < 0.0005
Pulse clarity	0.04 (0.01)	0.42 (0.05)	0.04 (0.01)	0.44 (0.03)	Welch’s *F*(3,9.89) = 486.68, *p* < 0.0005

*The average values of note density and pulse clarity (SD in brackets) for the ragas with calm, happy, sad and tensed experienced emotions are listed in the table.*

There were no outliers and the data for note density and pulse clarity was normally distributed for each group, as assessed by boxplot and Shapiro–Wilk test (*p* < 0.05), respectively. Since, homogeneity of variances, as assessed by Levene’s Test of Homogeneity of Variance was violated for both note density (*p* = 0.003) and pulse clarity (*p* = 0.02), the results of Welch ANOVA and *post hoc* Games-Howell test for multiple comparisons are reported.

The results of one-way ANOVA indicate that there were statistically significant differences in note densities, depending on the experienced emotion [Welch’s *F*(3,8.78) = 59.11, *p* < 0.0005). The note density was higher for *gat* of *ragas* rated as happy (*M* = 1.28, SD = 0.13) as compared to calm (*M* = 0.78, SD = 0.08) and for tensed (*M* = 1.18, SD = 0.11) as compared to sad (*M* = 0.73, SD = 0.02). Games-Howell *post hoc* analysis revealed that the mean increase of note density from *alaap* of *ragas* rated as ‘calm’ to *gat* of *ragas* rated as ‘happy’ [0.50, 95% CI (0.31,0.70)] was statistically significant (*p* < 0.005). Similarly, the increase from *alaap* of *ragas* rated as ‘sad’ to *gat* of *ragas* rated as ‘tensed’ [0.45, 95% CI (0.28,0.62), *p* = 0.001] was statistically significant (*p* < 0.005).

The results of one-way ANOVA for pulse clarity showed similar results, [Welch’s *F*(3,9.89) = 486.68, *p* < 0.0005]. The pulse clarity for *gat* of *ragas* rated as happy (*M* = 0.42, SD = 0.05) or tensed (*M* = 0.44, SD = 0.03) was higher as compared to *alaap* of *ragas* rated as calm (*M* = 0.04, SD = 0.01) or sad (*M* = 0.04, SD = 0.01). Games-Howell *post hoc* analysis revealed that the mean increase of pulse clarity from *alaap* of *ragas* rated as ‘calm’ to *gat* of *ragas* rated as ‘happy’ [0.38, 95% CI (0.31,0.45)] was statistically significant (*p* < 0.005). Similarly, the increase from *alaap* of *ragas* rated as ‘sad’ to *gat* of *ragas* rated as ‘tensed’ [0.40, 95% CI (0.31,0.45)] was statistically significant, (*p* < 0.005).

In summary, both tempo and rhythmic regularity of a *raga* modulate emotional response and high arousal emotions (happy and tensed) are associated with faster rhythm.

#### Effect of Tonality

To study the effect of tonality on emotional response, correlation and stepwise linear regression analysis was conducted. Since, listeners of *gat* of *ragas* experienced high arousal emotions; analysis was conducted only for *gat.*

**FIGURE 4:**
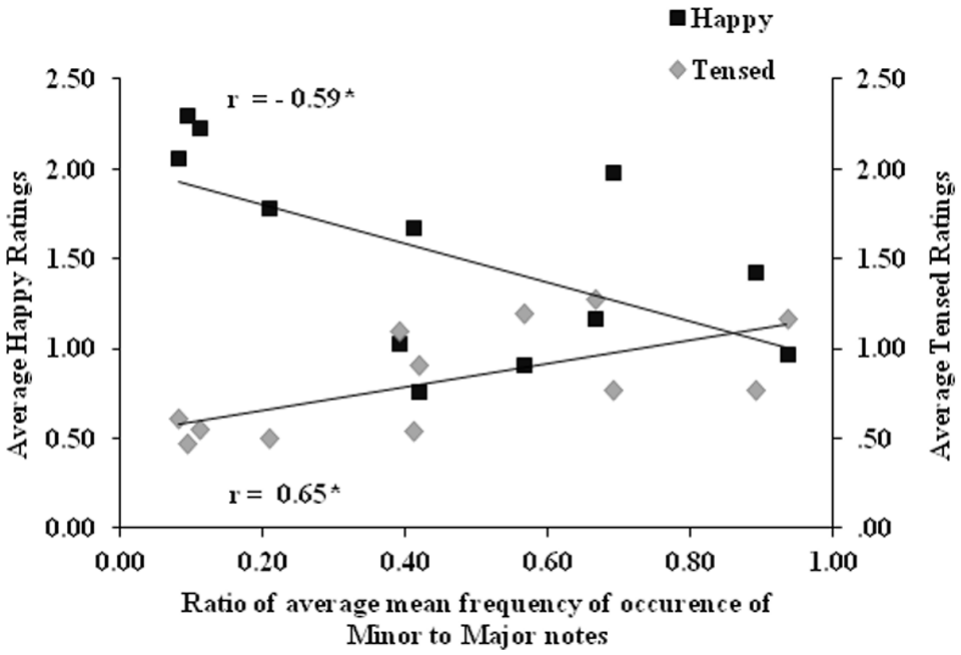
**Correlation plot between the ratio of mean frequency of occurrence of minor to major tonic intervals and average happy and tensed ratings across gat of *ragas***. The correlation coefficients (r) marked with asterisk (^∗^) are significant at *p* < 0.05.

To assess the influence of minor and major intervals on happy and tensed ratings, the ratio of mean frequency of occurrence of minor to major intervals was estimated and correlated with average ratings of ‘happy’ and ‘tensed’ emotion. The correlation plot of average ‘happy’ and ‘tensed’ ratings with the ratio of mean frequency of occurrence of minor to major tonic intervals is shown in **Figure 4**. The plot indicates that minor/major tonic interval frequency ratio is negatively correlated with happy ratings (*r* = -0.59, *p* < 0.05) and positively correlated with tensed ratings (*r* = 0.65, *p* < 0.05). This indicates that an increase in mean frequency of occurrence of minor intervals is associated with tense emotion, whereas an increase in mean frequency of occurrence of major intervals is associated with happy emotion.

Further, correlations were calculated between the average rating of an emotion and mean frequency of occurrence of each of the tonic intervals across the twelve *ragas* (refer to **Table 4**). The results of the correlation analysis indicate that the mean frequency of occurrence *komal re, shuddh Re,* and* komal dha* have significant correlations. To characterize which of these tonic intervals are the best subset for predicting the emotional response stepwise linear regression analysis was conducted. In the regression analysis, the vector containing average ratings for an emotion across the 12 *ragas* was taken as the dependent variable and the mean frequency of occurrence of the 12 tonic intervals were taken as the independent variables. The assumptions of linearity, independence of errors, homoscedasticity and normality of residuals were met. The results of regression analysis are reported in **Table 5**. The percent mean frequency of occurrence of the minor second (*komal re, re*) explained 58% of the variance for ratings of ‘happy’ emotion [*R*^2^ = 0.58, *F*(1,10) = 16.48, *p* < 0.05] and 89% of the variance for ratings of ‘tensed’ emotion [*R*^2^ = 0.89, *F*(1,10) = 44.46, *p* < 0.001]. The mean frequency of occurrence of *komal re* was significantly negatively correlated with ‘happy’ (β = -0.79, *p* < 0.05) and positively correlated with ‘tensed’ (β = 0.91, *p* < 0.001) emotion ratings.

**Table 4:** The table lists the correlation coefficients of correlations between the average emotion ratings and mean frequency of occurrence of each tonic interval across the 12 *ragas*.

	Happy	Romantic	Calm	Longing	Tensed	Sad
*Sa*	0.17	0.20	0.30	0.11	0.01	-0.12
*re*	-0.79^∗∗^	-0.83^∗∗^	-0.88^∗∗^	0.60^∗^	0.91^∗∗^	0.74^∗∗^
*Re*	0.66^∗^	0.66^∗^	0.59^∗^	-0.65^∗^	-0.67^∗^	-0.69^∗^
*ga*	0.05	-0.00	0.07	0.08	0.04	-0.06
*Ga*	0.49	0.52	0.45	-0.47	-0.57	-0.42
*Ma*	0.00	0.15	0.18	0.22	-0.14	0.16
*ma*	-0.47	-0.51	-0.66^∗^	0.20	0.47	0.35
*Pa*	0.25	0.08	-0.06	-0.22	0.14	-0.23
*dha*	-0.74^∗∗^	-0.67^∗^	-0.41	0.89^∗∗^	0.61^∗^	0.80^∗∗^
*Dha*	-0.13	-0.10	-0.12	-0.15	-0.00	0.04
*ni*	0.05	0.01	0. 20	0.16	-0.11	-0.03
*Ni*	0.08	0.05	-0.08	-0.39	-0.06	-0.19

*The correlation coefficients (β) marked with a single (^∗^) and a double asterisk (^∗∗^) are significant correlations at p < 0.05 and p < 0.001 respectively.*

**Table 5:** Results of Stepwise multiple linear regressions performed in order to determine the variance of the emotional responses explained by the tonic intervals in gat of *ragas*.

Happy	Romantic	Calm	Longing	Tensed	Sad
*re*, β = -0.79^∗^	*re*, β = -0.83^∗∗^	*re*, β = -0.88^∗∗^	*dha*, β = 0.89^∗∗^	*re*, β = 0.91^∗∗^	*dha*, β = 0.80^∗^
*R*^2^ = 0.58	*R*^2^ = 0.66	*R*^2^ = 0.75	*R*^2^ = 0.78	*R*^2^ = 0.89	*R*^2^ = 0.60
*F*(1,10) = 16.48, *p* < 0.05	*F*(1,10) = 22.19, *p* = 0.001	*F*(1,10) = 330.61, *p* < 0.001	*F*(1,10) = 39.22, *p* < 0.001	*F*(1,10) = 44.46, *p* < 0.001	*F*(1,10) = 17.23, *p* < 0.05


*The results of the first predictive model with values of adjusted R^2^, standardized coefficient (β) and F ratio of ANOVA results are reported in the table. The correlation coefficients (β) marked with a single (^∗^) and a double asterisk (^∗∗^) are significant correlations at p < 0.05 and p < 0.001 respectively.*

## Discussion and Conclusion

This study reports for the first time emotional responses of North Indian Classical *ragas* when rendered in two distinct presentation modes, namely, *alaap* and *gat*. Specifically, we found that (1) distinct emotional responses are associated with *alaap* and *gat* of a *raga*. (2) Pulse clarity and tempo significantly influenced the emotion experienced in terms of arousal. (3) Major intervals (*shuddh swaras*) are predictive of reported positive valence while minor intervals (*komal swaras*) are predictive of reported negative valence. (4) The minor second is a significant predictor of negative valenced emotional response. We discuss below the implications of these results.

### *Ragas* and Emotional Response

The key finding of our study was the experimental verification of the hypothesis that distinct emotional responses would be associated with *alaap* and *gat* of a *raga*. During the arrhythmic phase (*alaap*), an artist introduces the notes of the *raga* and the exposition is focused on setting the scale and the key structure of the melody. The rhythmic phase (*gat*) on the other hand, is faster and rhythmic and a percussionist accompanies the artist. As a consequence, the *alaap* of *raga* is believed to set the mood of *raga,* while *gat* enhances perception of emotion for that *raga* (Chib, 2004; Juslin and Sloboda, 2011).

Our results indicate that *ragas* evoke a gamut of responses that range from ‘happy’ and ‘calm’ to ‘tensed’ and ‘sad’ (as shown by results in **Figure 1**, Supplementary Tables S1 and S2). In particular, the emotional response to *ragas* (like *Desh* and *Tilak Kamod*) shifts from ‘calm/soothing’ in the slower arrhythmic* alaap* to ‘happy’ in the faster rhythmic *gat*. In parallel, the emotional response of ‘sad’ in the slower arrhythmic phase shifts to‘tensed’ in the faster rhythmic phase (e.g.,* Shree* and *Miyan ki Todi*; **Figures 1A,B**). An interesting feature was the fact that all *ragas* universally generated a calming effect and anger remained the lowest rated emotion category. This is in consensus with research on Western music which shows that negative emotions like anger which are regularly experienced in everyday life are only rarely experienced in response to music (Juslin and Laukka, 2004; Laukka, 2006). However, anger relates to irritation, which is most likely to arise when people are exposed to music they fail to understand, dislike, or even abhor (Zentner et al., 2008), or when the music is unwanted/too loud and thus considered annoying, i.e., noise.

### Role of Musical Structure in Emotion Experienced

The results revealed that pulse clarity (estimate of rhythmic regularity) and note density (estimate of tempo) differ among *ragas* with different experienced emotions, where high arousal emotions (happy/tensed) are associated with a faster rhythm. In addition, tonality significantly influenced the emotion experienced as the increase in mean frequency of occurrence of minor intervals was associated with ‘tensed’ emotion whereas increase in mean frequency of occurrence of major intervals was associated with ‘happy’ emotion (refer to **Figure 4**). Thus, our results indicated that the tonal distribution patterns determine the underlying mood (*rasa*) of a *raga* and the presence of rhythm changes the level of arousal of emotions experienced.

These results appear to be universal across musical genres. For instance, in a study conducted by Husain et al. (2002), participants were asked to listen to four versions of Mozart sonata (fast-major, fast-minor, slow-major, and slow-minor) and rate their affective state on adjectives on vigor-activity subscale describing high arousal (lively, active, energetic, full of pep, and vigorous) and depression-dejection subscale describing negative mood (sad, unworthy, discouraged, lonely, and gloomy). They found that the music manipulations were associated with changes in arousal and mood. The fast-tempo versions were accompanied by increases in listeners’ levels of arousal, whereas the slow-tempo versions caused decreases in arousal. By contrast, the mode of the piece was associated with listeners moods. Those who heard the major mode became more positive in mood, whereas the minor mode caused negative shifts in mood. Thus, tempo and mode were relatively separable in this regard. Another study by Laukka and Gabrielsson (2000) showed that rhythm alone can convey emotions. This tested the idea that drumbeats could express emotions by playing clips of drum performances and found that listeners could accurately indicate which emotions the drummers were attempting to express even though the drummers were limited in the instruments and rhythms they could utilize. While our results clearly support the idea that rhythm plays a significant role in emotional response to music, with the existing design we are unable to separate the specific roles played by tempo and rhythmic regularity and merit further research.

Tonality analysis of *ragas* revealed that *ragas* with positive valence (for e.g., calm and happy) have a greater mean frequency of occurrence of major intervals (*shuddh swaras*) where as *ragas* with negative valence (for e.g., sad or tensed) are characterized by an increased frequency of minor intervals (*Komal swaras*; **Figure 3**). Within the subset of *ragas* used in this study, there was a significant difference in the mean frequency of occurrence of minor second (*komal re*), major second (*shuddh Re*), major third (*shuddh Ga*), and minor sixth (*komal dha*; refer to **Figure 3**). The mean frequency of occurrence of minor second shows significant negative correlation with‘happy,’ ‘romantic,’ and ‘calm’ experienced emotions which suggests that its absence plays an important role in the rating of a *raga* as positive. On the other hand, it shows significant positive correlation with ‘sad’ and ‘tensed’ experienced emotions (refer to **Table 4**). In addition, the minor second appears as a significant positive predictor of ‘tensed’ emotion for *gat* of *ragas* and explains 89% of variance in ratings of ‘tensed’ emotion (refer to **Table 5**). Tonality, by definition, creates a hierarchical system in which the ‘minor second’ is a significant ‘pointer’ to the tonic, a ‘leading note.’ In tonal music therefore, the minor second holds a position as an ‘upper leading note’ (Moore, 2014). However, it is a dissonant interval, since the semitone overtone relationship is 16/15 (**Table 1**). By definition, for a consonant interval, the interval between two notes is a simple numerical ratio of frequencies in terms of the harmonic overtone series (Plomp and Levelt, 1965). Based on structure and composition, all *ragas* are tonal and the tonic is the reference point. As suggested by Moore, the ‘minor second’ with its tension and high ‘yearning’ toward the tonic, may build a narrative of hope or fear, the resolution of which brings associations of tension, yearning and a release of energy. The results of this study encourage us to hypothesize that minor second in NICM plays an important role in conveying tension and further studies should attempt to investigate its role in detail.

At the same time, the present study is characterized by certain limitations which restrict the generalizability of these findings. The first of these is with regard to the concept of *sruti* in NICM. The 12-tone semitone system of western music is clearly at odds with the 22 *sruti* system since some semitones are composed of one *sruti* while others of two or more. However, the *sruti* system still cannot account for the minute deviations from the norm, many of which are unconsciously presented by the artist. Thus for the purposes of this study, we were obliged to use the twelve semitone system, while making allowances for minor variations which is a limitation of this study. Secondly, to arrive at an emotion label for a *raga*, we should ideally have multiple excerpts of the same *raga*, played on different instruments by different performers (in *alaap* or *gat*) and then rated by listeners. When responses across different performers and different intruments all emerge with the same label, we would then have truly assigned an emotion label to a *raga*. Hopefully, further studies conducted on a large scale can address this question. Finally, we used self-reports to assess participants emotional responses. It can therefore not be ruled out that at least some of the participants rated expressed emotion instead of experienced emotion.

Nevertheless, our study provides new evidence that *ragas* evoke distinct emotional responses across distinct presentation modes (*alaap* and *gat*). This opens up the possibility of using different *ragas* as robust mood-inducing stimuli, which is relevant for studies on emotion. We also found that rhythmic regularity and tempo influence emotion experienced. Finally, one of the most interesting findings of our study was the association of the minor second with ‘tensed’ emotion. This is distinct from past work in Western classical music that has shown an association for the minor third with sadness in Western music (Curtis and Bharucha, 2010). Future work will attempt to extend these findings to larger population in order to delineate influences of culture, familiarity and musical training on emotion experienced.

## Conflict of Interest Statement

The authors declare that the research was conducted in the absence of any commercial or financial relationships that could be construed as a potential conflict of interest.
