# Supplementary material for: Emotional responses to Hindustani raga music: the role of musical structure
Source: Front Psychol. 2015 Apr 30;6:513. doi: 10.3389/fpsyg.2015.00513 (PMC4415143; doi:10.3389/fpsyg.2015.00513)
Supplement: Supplementary file 1 [file Data_Sheet_1.DOCX]

***Supplementary Material***

## Emotional responses to Hindustani *Raga* music: The role of musical structure

Avantika Mathur^1^, Suhas H. Vijayakumar^1^, Bhismadev Chakrabarti^2^ and Nandini C. Singh^1^.

^1^Speech and Language Laboratory, National Brain Research Centre, Nainwal Mode, Manesar

122 050, Haryana, India

^2^Centre for Integrative Neuroscience and Neurodynamics, School of Psychology and Clinical Language Sciences, University of Reading, Reading RG6 6AL, UK

Corresponding Author:

Dr. Nandini C. Singh

Speech and Language Laboratory, National Brain Research Centre, Nainwal Mode, Manesar

122 050, Haryana, India

Email – nandini@nbrc.ac.in

1. Supplementary Tables

Table S1

Median of ratings of each emotion for *alaap* of all the *ragas*.

| S.No. | *Raga* | Happy | Romantic/Tender | Devotional | Calm/ Soothed | Angry | Longing/ Yearning | Tensed/ Restless | Sad | Chi-Square |  | W | |
| --- | --- | --- | --- | --- | --- | --- | --- | --- | --- | --- | --- | --- | --- |
| 1 | *Hamsadhwani* | 1.00 | 1.00 | 1.00 | **2.00** | 0.00 | 1.00 | 0.00 | 1.00 | χ2 (7, N=109) = | 228.23 | .299 | |
| 2 | *Tilak Kamod* | 1.00 | 1.00 | 1.00 | **2.00** | 0.00 | 1.00 | 0.00 | 1.00 | χ2 (7, N=109) = | 223.80 | .293 | |
| 3 | *Desh* | 1.00 | 1.00 | 1.00 | **2.00** | 0.00 | 1.00 | 0.00 | 1.00 | χ2 (7, N=120) = | 271.21 | .323 | |
| 4 | *Rageshree* | 1.00 | 1.00 | 1.00 | **2.00** | 0.00 | 1.00 | 0.00 | 1.00 | χ2 (7, N=120) = | 230.60 | .275 | |
| 5 | *Jog* | 0.00 | 1.00 | 1.00 | **2.00*** | 0.00 | **2.00*** | 0.00 | 1.50* | χ2 (7, N=120) = | 203.83 | .243 | |
| 6 | *Yaman* | 0.00 | 1.00 | 1.00 | **2.00** | 0.00 | 1.00* | 0.00 | 1.00* | χ2 (7, N=108) = | 207.16 | .274 | |
| 7 | *Malkauns* | 0.00 | 1.00 | 1.00 | **2.00*** | 0.00 | **2.00*** | 0.00 | 1.00* | χ2 (7, N=120) = | 254.47 | .303 | |
| 8 | *Marwa* | 0.00 | 0.00 | 1.00 | 1.00 | 0.00 | **2.00*** | 1.00 | **2.00*** | χ2 (7, N=110) = | 292.31 | .380 | |
| 9 | *Basant Mukhari* | 0.00 | 0.00 | 1.00 | 1.50* | 0.00 | **2.00*** | 0.00 | **2.00*** | χ2 (7, N=120) = | 286.85 | 0.341 | |
| 10 | *Lalit* | 0.00 | 0.00 | 1.00 | **2.00*** | 0.00 | **2.00*** | 0.00 | **2.00*** | χ2 (7, N=120) = | 267.72 | .319 | |
| 11 | *Shree* | 0.00 | 0.00 | 1.00 | 1.00* | 0.00 | **2.00*** | 1.00 | **2.00*** | χ2 (7, N=108) = | 241.26 | .319 | |
| 12 | *Miyan ki Todi* | 0.00 | 0.00 | 1.00 | 1.50* | 0.00 | 1.50* | 1.00 | **2.00** | χ2 (7, N=108) = | 240.22 | .318 | |
|  |  |  |  |  |  |  |  |  |  |  |  |  |  |

*Note.* The Friedman one way analysis of variance by ranks was conducted to evaluate differences in medians of ratings of emotions for each *raga*. The test was significant for all the *ragas* at p < 0.001. The value of chi-square and Kendall’s coefficient of concordance **(W)** are given for each *raga*. Follow-up pairwise comparisons were conducted using a Wilcoxon test controlling for the Type I errors across the ratings of emotions at 0.001 level using the Holm’s Sequential Bonferroni method.

The median ratings that did not differ significantly from each other (p > 0.001) are marked with an asterisk (*).

Table S2

Median of ratings of each emotion for *gat* of all the *ragas*.

| S.No. | *Raga* | Happy | Romantic/Tender | Devotional | Calm/ Soothed | Angry | Longing/ Yearning | Tensed/ Restless | Sad | Chi-Square |  | W |
| --- | --- | --- | --- | --- | --- | --- | --- | --- | --- | --- | --- | --- |
| 1 | *Hamsadhwani* | **2.00*** | **2.00*** | 1.00 | **2.00*** | 0.00 | 1.00 | 0.00 | 0.00 | χ2 (7, N=122) = | 389.80 | .456 |
| 2 | *Tilak Kamod* | **2.00*** | **2.00*** | 1.00 | **2.00*** | 0.00 | 1.00 | 0.00 | 0.00 | χ2 (7, N=122) = | 323.55 | .379 |
| 3 | *Desh* | **2.00*** | **2.00*** | 1.00 | **2.00*** | 0.00 | 1.00 | 0.00 | 0.00 | χ2 (7, N=109) = | 300.87 | .394 |
| 4 | *Rageshree* | **2.00*** | 1.00* | 1.00* | **2.00*** | 0.00 | 1.00* | 0.00 | 0.00 | χ2 (7, N=109) = | 225.38 | .295 |
| 5 | *Jog* | **2.00*** | 1.00 | 1.00 | **2.00*** | 0.00 | 1.00 | 0.00 | 0.00 | χ2 (7, N=108) = | 202.31 | .268 |
| 6 | *Yaman* | **2.00*** | 1.00 | 1.00 | **2.00*** | 0.00 | 1.00 | 0.00 | 0.00 | χ2 (7, N=123) = | 257.21 | .299 |
| 7 | *Malkauns* | 1.00 | 1.00 | 1.00* | **2.00*** | 0.00 | 1.00* | 0.00 | 1.00 | χ2 (7, N=110) = | 162.55 | .211 |
| 8 | *Marwa* | 1.00* | 1.00 | 1.00 | 1.00* | 0.00 | 1.00* | 0.00 | 1.00* | χ2 (7, N=122) = | 66.38 | .078 |
| 9 | *Basant Mukhari* | 1.00 | 1.00 | 1.00 | 1.00* | 0.00 | **2.00*** | 1.00* | 1.00* | χ2 (7, N=109) = | 109.64 | .144 |
| 10 | *Lalit* | 0.00 | 1.00 | 1.00 | **2.00*** | 0.00 | **2.00*** | 0.00 | 1.50* | χ2 (7, N=108) = | 133.55 | .177 |
| 11 | *Shree* | 1.00* | 1.00 | 1.00* | 1.00* | 0.00 | 1.00* | 1.00* | 1.00* | χ2 (7, N=121) = | 72.33 | .085 |
| 12 | *Miyan ki Todi* | 1.00* | 0.00 | 1.00* | 1.00* | 0.00 | 1.00* | 1.00* | 1.00* | χ2 (7, N=121) = | 91.75 | .108 |

*Note.* A Friedman one way analysis of variance by ranks was conducted in order to evaluate the differences in medians of ratings of emotions for each *raga*. The test was significant for all the *ragas* at p < 0.001. The value of chi-square and kendall's coefficient of concordance (W) are given for each *raga*. Follow-up pairwise comparisons were conducted using a Wilcoxon test controlling for the Type I errors across the ratings of emotions at 0.001 level using the Holm’s Sequential Bonferroni method.

The median ratings that did not differ significantly from each other (p > 0.001) are marked with an asterisk (*).

Table S3

The table lists the results of the two-tailed Mann-Whitney U test conducted to assess the differences in the mean frequency of occurrence of major notes (*shuddh swaras*, represented by capital letters) and minor notes (*komal swaras*, represented by small letters) for statistical significance in *ragas* with ‘calm’ and ‘sad’ emotional response.

| Interval Size | Notes | *Raga* | N | Mean | Mean Rank | Sum of Ranks | U | Z | p |
| --- | --- | --- | --- | --- | --- | --- | --- | --- | --- |
| 0 | *Sa* | Calm | 6 | 20.49 | 5.00 | 30.00 | 9.000 | -1.44 | .15 |
|  |  | Sad | 6 | 23.85 | 8.00 | 48.00 |  |  |  |
| 100* | *re* | Calm | 6 | 0.60 | 3.50 | 21.00 | 0.000 | -2.88 | .001 |
|  |  | Sad | 6 | 10.39 | 9.50 | 57.00 |  |  |  |
| 200* | *Re* | Calm | 6 | 9.91 | 8.50 | 51.00 | 6.000 | -1.92 | .05 |
|  |  | Sad | 6 | 1.53 | 4.50 | 27.00 |  |  |  |
| 300 | *ga* | Calm | 6 | 3.16 | 7.33 | 44.00 | 13.000 | -0.80 | .42 |
|  |  | Sad | 6 | 5.88 | 5.67 | 34.00 |  |  |  |
| 400* | *Ga* | Calm | 6 | 17.84 | 8.83 | 53.00 | 4.000 | -2.24 | .02 |
|  |  | Sad | 6 | 8.97 | 4.17 | 25.00 |  |  |  |
| 500 | *Ma* | Calm | 6 | 9.19 | 7.00 | 42.00 | 15.000 | -0.48 | .63 |
|  |  | Sad | 6 | 8.06 | 6.00 | 36.00 |  |  |  |
| 600 | *ma* | Calm | 6 | 3.70 | 5.50 | 33.00 | 12.000 | -0.96 | .34 |
|  |  | Sad | 6 | 7.03 | 7.50 | 45.00 |  |  |  |
| 700 | *Pa* | Calm | 6 | 6.07 | 7.17 | 43.00 | 14.000 | -0.64 | .52 |
|  |  | Sad | 6 | 4.16 | 5.83 | 35.00 |  |  |  |
| 800* | *dha* | Calm | 6 | 0.35 | 3.50 | 21.00 | 0.000 | -2.88 | .001 |
|  |  | Sad | 6 | 8.35 | 9.50 | 57.00 |  |  |  |
| 900 | *Dha* | Calm | 6 | 5.77 | 6.83 | 41.00 | 16.000 | -0.32 | .75 |
|  |  | Sad | 6 | 4.37 | 6.17 | 37.00 |  |  |  |
| 1000 | *ni* | Calm | 6 | 9.22 | 7.17 | 43.00 | 14.000 | -0.64 | .52 |
|  |  | Sad | 6 | 7.17 | 5.83 | 35.00 |  |  |  |
| 1100 | *Ni* | Calm | 6 | 13.71 | 7.17 | 43.00 | 14.000 | -0.64 | .52 |
|  |  | Sad | 6 | 10.25 | 5.83 | 35.00 |  |  |  |

*Note.* The mean frequency of occurrence of major second (*shuddh Re*) (z = -1.92, p ≤ 0.05) and major third (*shuddh Ga*) (z = -2.24, p < 0.05) was significantly higher in *ragas* with the ‘calm’ emotional response .The mean frequency of occurrence of *komal re* (z = -2.88, p < 0.05) and *komal dha* (z = -2.88, p < 0.05) was significantly higher in *ragas* with the ‘sad’ emotional response.

Asterisks indicate statistically significant differences between the underlying distributions (* p < 0.05).

# Supplementary Figure legend

*Supplementary Figure 1.* The histogram depicting the pitch class distributions across the three octaves for *ragas* with ‘calm’ (red) and ‘sad’ (blue) emotional response.

*Supplementary Figure 2.* The tonic intervals of *ragas*. The percent mean frequency of occurrence of tonic intervals averaged across *gat* of *ragas* for which emotional response was ‘happy’ and ‘tensed/longing’. Two-tailed Mann Whitney U-test showed that percent mean frequency of occurrence of *komal re* and *komal dha* were significantly higher for *ragas* with ‘tensed/longing’ emotional response at p < 0.05 (marked with an asterisk (*)). In addition, percent mean frequency of occurrence of *shuddh Re* and *shuddh Ga* were significantly higher for *ragas* with ‘happy’ emotional response at p < 0.05 (marked with an asterisk (*)).

# Supplementary Audio clips

**Audio clip S1.** Exemplar of positive *raga* (*Desh*) played in *alaap.*

**Audio clip S2.** Exemplar of plaintive *raga* (*Basant Mukhari*) played in *alaap*.

**Audio clip S3.** Exemplar of positive *raga* (*Desh*) played in *gat.*

**Audio clip S4.** Exemplar of plaintive *raga* (*Basant Mukhari*) played in *gat*.
