# Supplementary figures and images for: Emotional responses to Hindustani raga music: the role of musical structure
Source: Front Psychol. 2015 Apr 30;6:513. doi: 10.3389/fpsyg.2015.00513 (PMC4415143; doi:10.3389/fpsyg.2015.00513)

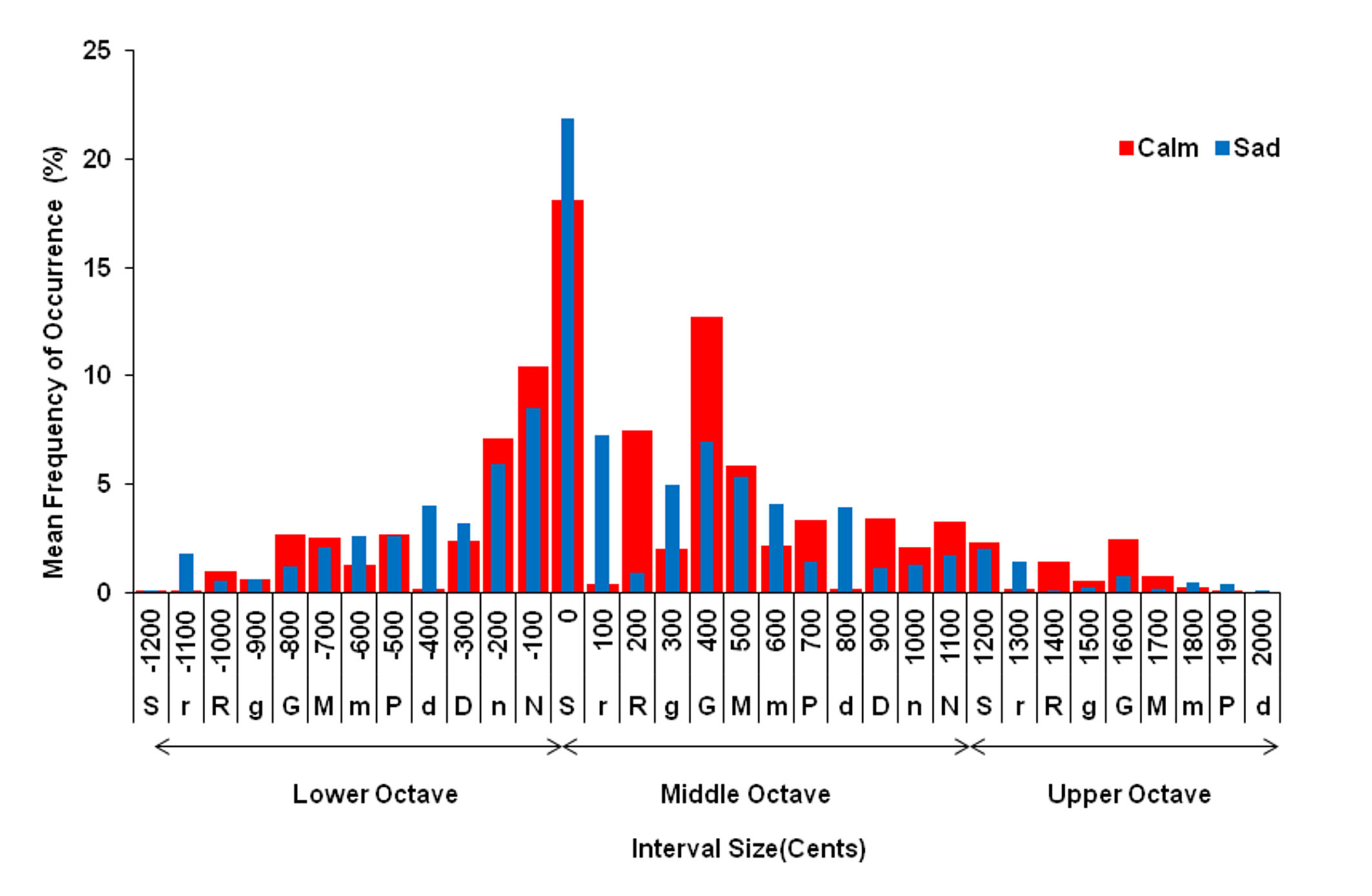

Supplement: Supplementary file 6 [file Image_1.TIF]

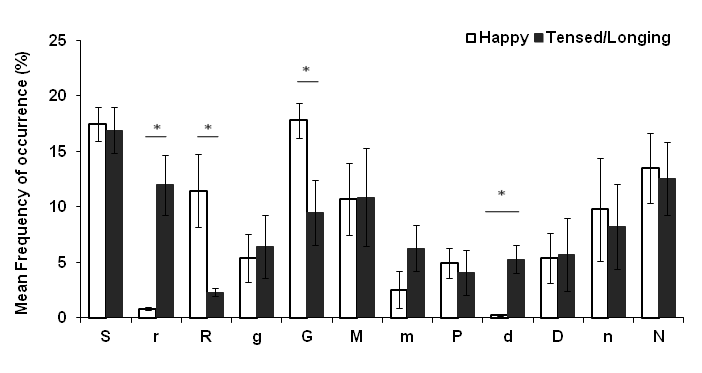

Supplement: Supplementary file 7 [file Image_2.TIF]
